# Will Crystal Parkin Help in Understanding the Future of Parkinson’s Disease?

**DOI:** 10.3389/fneur.2015.00035

**Published:** 2015-02-24

**Authors:** Diana Angelika Olszewska, Tim Lynch

**Affiliations:** ^1^Department of Neurology, Dublin Neurological Institute, Mater Misericordiae University Hospital, Dublin, Ireland

**Keywords:** parkinsonism, crystal structure, crystal parkin, RING structure, E3 ligase

Parkinson’s disease (PD) is the second most common neurodegenerative disorder following Alzheimer’s disease. Mutations in several genes are reported to cause Parkinson’s disease (SNCA, LRRK2, VPS35, Parkin, PINK1, DJ-1) (1). Parkin mutations are responsible for 50% of autosomal recessive juvenile PD and 15% of young onset (<age of 45) sporadic PD (1). Parkin has a neuroprotective role (2) by eliminating damaged mitochondria and reactive oxygen species through autophagy (mitophagy). Parkin mutations cause deregulation of the mitochondrial homeostasis mechanism and subsequent accumulation of the impaired mitochondria and have a role in cell surface signaling and tumor suppression (3, 4).

Neuroprotective therapy development in PD has been frustratingly slow, partially due to the lack of knowledge of the precise structure of potential target molecules, such as alpha-synuclein, LRRK2, or Parkin. Structure-based drug design is reliant upon identifying the crystal structure of the target molecule. For example, enhancing Parkin housekeeping ability could stop accumulation of the reactive oxygen species and subsequent neurodegeneration.

Johnston’s group recently published in Nature Communications the first crystal structure of Parkin and described the molecule at the atomic level (5). Parkin is a canonical RING-between-RING (RBR) E3 ligase (Figure 1). E3 ligases have a crucial role in the final step of ubiquitination by transferring ubiquitin from E2 enzyme to lysine substrate (6). Studies suggest that RBR ligases may contain a catalytic cysteine residue, similarly to HECT ligases (E3 enzymes influencing specificity of ubiquitylation). The organization of domains, exact residues, and regulation of ligase activity remains elusive. Parkin is activated through phosphorylation of ubiquitin by PINK1 (do not forget that mutations in PINK1 are also associated with autosomal recessive PD). Parkin is a folded-in-half molecule consisting of ubiquitin-like domain (UBL), followed by four RING domains, each bonded to two Zn atoms (3) (Figure 1). RING 0 (also called a unique Parkin domain-UPD) (4) at the N-terminal end is an innovative structure resembling zinc-finger domain. RING1-IBR (in between ring) domains are spatially opposite RING2. Parkin has a unique RING0:RING2 (C-terminal) bond which may alter Parkin’s active control site demonstrated by mass spectrometry to be at catalytic cysteine 431 (C431) (Figure 1).

**Figure 1:**
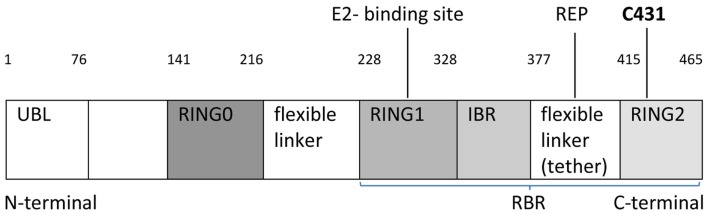
**Schematic linear structure of Parkin, showing UBL (ubiquitin-like domain) followed by four RING domains: RING0, RING1, IBR (in between ring), RING2 with two linkers (IBR-RING2 linker referred to as tether) indicating an area of potential structural flexibility, E2-binding site, REP (repressor element), C431 active site residue and protein residues (1–465), RBR: Ring-between-Ring structure**.

Simultaneously, Trempe et al. (2) confirmed these findings and similarly described how RING0 occludes the active site (C431) in RING2, while a flexible IBR-R2 linker (tether) residue (repressor element of Parkin-REP or W403) sits in a pocket on RING1 and may anchor the tether to RING1 and block the E2-binding site (Figure 1). The interface has two compact domains separated by two linkers: (1) RING1–IBR containing E2-binding site and (2) RING0–RING2 with the C431 active site (5).

The discovery of the crystal structure of Parkin provides new opportunities for defining (1) the molecular function of Parkin’s target site, (2) the binding of possible therapeutic particles, (3) the mechanism of action of Parkin, and (4) the modulation/enhancement of novel drug molecules (7). For example, mutations of the C431 active site cause loss of the Parkin mitochondrial degradation mechanism (5), whereas deletion of RING0 (lack of occlusion of C431 as described) causes increased C431 reactivity and Parkin autoubiquitination (3). Deletion of UBL domain with/without the linker has little effect on Parkin activity (2). Mutations in hydrophobic residues of RING0:RING2 interface increase autoubiquitination (5).

Crystal structure research and discovery has increased our understanding about lysozyme function, vitamin B12 action, penicillin, and its modifications (7, 8) and has been used in the discovery of the new treatments for glaucoma (dorzolamide), HIV (protease inhibitors: saquinavir, indinavir, ritonavir, and nelfinavir) (9), and chronic myelogenous leukemia (tyrosinase-kinase inhibitor- Imatinib) (10). It has also clarified the mechanism of leukemic drug-resistance (11) and is currently relevant to HIV1 research with the development of reverse transcriptase capable of the rapid crystallization, which will help the design of new anti-AIDS medications (12).

In summary, Johnston group have improved our understanding of the Parkin structure at the atomic level with the discovery of its crystal structure. Mapping of Parkin mutations affecting the stability of the molecule (mutations in Zn), catalytic reactions (active site at C431), and the interactions among domains (E2-binding site) (3, 4) may help future drug development in PD and neurodegeneration by enhancing activity at the active site or by understanding further its molecular interactions.

## Author Contributions

DO: writing first draft and corrections. TL: review and critique.

## Conflict of Interest Statement

The authors declare that the research was conducted in the absence of any commercial or financial relationships that could be construed as a potential conflict of interest.
